# Shortening 7T MP2RAGE acquisition with compressed sensing: Evaluating quantitative accuracy and structural consistency

**DOI:** 10.1371/journal.pone.0325783

**Published:** 2025-06-16

**Authors:** Ikuhiro Kida

**Affiliations:** 1 Center for Information and Neural Networks, National Institute of Information and Communications Technology, and Osaka University, Suita, Osaka, Japan; Jikei University School of Medicine, JAPAN

## Abstract

The aim of this study was to systematically evaluate the impact of compressed sensing (CS) on acquisition time, image quality, T_1_ mapping accuracy, and segmentation consistency in magnetization-prepared 2 rapid acquisition gradient echo (MP2RAGE) at ultra-high fields (UHF). MP2RAGE sequences were acquired using the CS and parallel imaging (PI) technique, i.e., generalized autocalibrating partially parallel acquisitions (GRAPPA), with varying undersampling factors and samples per repetition time (TR). The acquisition time, quantitative accuracy of T_1_ mapping, and segmentation consistency across regions of interest (ROIs) were assessed. CS-MP2RAGE achieved a 61% reduction in acquisition time (< 3 min) compared with PI-MP2RAGE and maintained comparable image quality, segmentation accuracy, and T_1_-mapping fidelity. Higher undersampling factors effectively reduced scan duration but introduced segmentation volume mismatches of up to 20% and increased T_1_ values, despite the images appearing similar to PI-MP2RAGE. Reducing the number of samples per TR enhanced image quality, allowing for higher undersampling factors without a significant loss of fidelity, a finding consistent with previous studies. However, excessively low sampling densities destabilized reconstruction in complex ROIs. Our findings demonstrate that CS-MP2RAGE significantly reduces scan time while maintaining high image quality and quantitative accuracy, making it a viable alternative to GRAPPA in UHF applications. The interplay between undersampling factors and samples per TR is crucial for optimizing scan efficiency. Future studies should explore its application in clinical and research settings.

## Introduction

Magnetic resonance imaging (MRI) is an essential technique in clinical diagnosis and research, including neurodegenerative diseases, brain morphometry, and multiple sclerosis lesion analysis, since it provides contrast and anatomical details of soft tissues. As a method of acquiring T_1_-weighted images used in MRI, the magnetization-prepared rapid acquisition gradient echo (MPRAGE) method is widely used at magnetic field strengths up to 3 Tesla. However, the magnetization-prepared 2 rapid acquisition gradient echo (MP2RAGE) method is considered suitable at magnetic field strengths higher than 3 Tesla.

MP2RAGE is particularly advantageous at ultra-high magnetic field strengths (> 7 Tesla) because it effectively reduces B_1_^+^ inhomogeneities and provides uniform T_1_-weighted contrast. At higher magnetic fields, B_1_^+^ field inhomogeneity and increased T_1_ relaxation times can significantly impair image quality in conventional T_1_-weighted imaging. MP2RAGE overcomes these challenges by combining two inversion-recovery gradient-echo images acquired at different inversion times into a composite image that is less sensitive to B_1_^+^ variations and proton density. This makes MP2RAGE especially valuable for anatomical imaging and T_1_ mapping at ultra-high field (UHF) strengths [1].

The use of UHF MRI systems offers significant advantages, such as an enhanced signal-to-noise ratio and increased spatial resolution, facilitating the detailed capture of fine anatomy. However, UHF MRI presents unique challenges, particularly increased B_1_ field inhomogeneity and susceptibility artifacts [1]. Despite its advantages, MP2RAGE imaging requires relatively long scan times, potentially limiting its clinical utility, especially in high-throughput environments and for patients who cannot tolerate prolonged imaging procedures. It may be time-limited in neuroscience applications. For example, the standard MP2RAGE protocol requires 7–10 minutes to acquire a single high-resolution dataset, even with parallel imaging acceleration [1–3], posing challenges regarding patient comfort and motion artifacts. Reducing scan time is essential for improving patient comfort, minimizing motion artifacts, and incorporating MP2RAGE into routine clinical and experimental workflows. Therefore, there is an urgent need to develop methods to reduce acquisition time without compromising image quality or diagnostic reliability.

Parallel imaging (PI) techniques, such as Sensitivity Encoding (SENSE) and generalized auto-calibrating partially parallel acquisitions (GRAPPA), enable undersampling factors of 2–3 times in MP2RAGE [1]. The performance of GRAPPA is constrained by the requirements for noise amplification and coil density, which may limit its effectiveness for higher undersampling factors. Recent advances, such as compressed sensing (CS), provide a promising solution to reduce scan time by up to 50% in various imaging modalities, such as cardiac MRI and angiography, by exploiting data sparsity to reconstruct high-quality images from undersampled datasets [4,5]. It has been demonstrated that a 57% time reduction is possible when CS is applied to MP2RAGE in 3 Tesla MRI [6], where whole-brain imaging can be performed in less than 4 min with little loss in image quality or diagnostic metrics.

Although CS algorithms rely on sparsity assumptions to recover missing data, excessive undersampling undermines these assumptions, causing artifacts such as ringing, blurring, and ghosting [6,7]. Furthermore, undersampling inherently reduces the acquired signal, resulting in a lower SNR. As the noise levels remain constant while the signal decreases, the reconstructed images become grainier, leading to a loss of structural details. Despite these advances, there is still a need to systematically evaluate these methods within the framework of MP2RAGE, particularly at UHF strengths, although a comparison has been conducted at 3 Tesla [6,8]. How well CS alone can outperform conventional parallel imaging in terms of acceleration and image quality at 7 Tesla remains an important question. In other words, quantitative metrics such as T_1_ mapping accuracy, segmentation consistency, and contrast-preserving properties require further validation across a broader range of datasets and settings.

This study aimed to systematically evaluate the effectiveness of compressed sensing in MP2RAGE in reducing acquisition time at 7 Tesla while preserving image quality and to determine the optimal approach to accelerate MP2RAGE acquisition by comparing the CS and PI techniques. In particular, this study evaluated the performance using quantitative metrics, such as the T_1_ relaxation time and segmentation accuracy in healthy individuals. The findings of this study provide novel insights into the actual deployment of the MP2RAGE protocol and will facilitate its integration into time-constrained clinical and research environments.

## Materials and methods

### Subjects

Data were collected from sixteen healthy participants (mean age: 22.6 ± 2.1 years; 7 males, 9 females). Recruitment of participants began on 29 August 2024 and ended on 14 January 2025. Informed consent was obtained in writing from all participants before the beginning of the experiment, and the study protocol was approved by the Ethics Committees of the National Institute of Information and Communications Technology (approval number: N230242304).

### MRI measurements

MRI was performed using a 7T whole-body MRI scanner (Siemens Magnetom Terra, Siemens Healthineers, Erlangen, Germany) with a 32-channel head coil. The MP2RAGE sequence, vendor-supplied “works in progress” packages, was employed with the following common acquisition parameters: spatial resolution = 1.0 mm isotropic voxel, field of view = 256 × 256 mm, 208 slices, repetition time (TR) = 5,000 ms, inversion times (TI1, TI2) = 800 ms and 2,600 ms, and flip angles (TI1, TI2) = 4° and 5°. For the PI-MP2RAGE images as a control, an undersampling factor of R = 3 was applied in the primary phase-encoding direction, with the partial Fourier set to 6/8, resulting in a total acquisition time of 7 min 17 s. These parameters are based on those used in a previous study [2,3]. In CS-MP2RAGE, the undersampling factor represents the degree to which k-space data acquisition is reduced relative to fully sampled imaging, thereby reflecting the extent of scan time acceleration. For instance, an undersampling factor of 8.0 corresponds to the acquisition of 12.5% of the full k-space data, achieving an eightfold reduction in acquisition time. In the present study, undersampling factors of 3.8, 4.4, 8.0, and 10.0 were employed, with the number of samples per TR adjusted accordingly to achieve the target acceleration rates (Table 1). The number of samples per TR indicates the number of phase-encoding lines acquired within a single TR of the MP2RAGE sequence. In conventional 3D MRI, the total number of acquired samples is determined by the product of the phase-encoding and slice-encoding matrix dimensions (e.g., Ny × Nz); however, the CS-MP2RAGE sequence utilized here reorganizes the acquisition using a Cartesian phyllotaxis trajectory with flexible grouping of samples per TR, as described by Mussard et al. (2020) [6]. The total number of acquired samples is calculated by applying the undersampling factor to the elliptically masked region of k-space rather than to the entire matrix. K-space data were sampled in an arc-wise manner according to a jittered Cartesian phyllotaxis pattern. This trajectory enables a smooth and continuous traversal of k-space in a quasi-spiral fashion, thereby minimizing abrupt changes in the imaging gradients and promoting temporal incoherence, both of which are essential for effective compressed sensing reconstruction. A detailed description of the CS-MP2RAGE with Cartesian phyllotaxis sampling and arc-wise ordering strategies can be found in Mussard et al. (2020) [6], to which readers are referred for further methodological details. Total acquisition time was shortened to 35% –76% of that of PI-MP2RAGE. MP2RAGE produces output images, including T_1_-weighted images termed uniform (UNI), denoised T_1_-weighted images termed UNI-DEN, and a T_1_ map, by combining two different gradient echo images with two different inversion times.

**Table 1:** Acquisition parameters for MP2RAGE with parallel imaging (PI) and compressed sensing (CS).

	*Participants*	*Undersampling factor*	*Samples/TR*	*TE* *(ms)*	*Total acquisition time* *(min:sec)*	*Reduction (%)*
*PI*	16/16			1.91	7:17	
*CS*	16/16	3.8	250	1.89	3:44	49%
*CS*	16/16	4.4	252	1.89	3:09	57%
*CS*	16/16	8.0	253	1.89	1:44	76%
*CS*	6/16	3.8	196	1.89	4:44	35%
*CS*	6/16	8.0	192	1.89	2:19	68%
*CS*	6/16	8.0	126	1.89	3:29	52%
*CS*	6/16	10.0	126	1.89	2:49	61%

### Analysis

The CS-MP2RAGE UNI-DEN images were co-registered to the PI-MP2RAGE space using a six-degree-of-freedom rigid body co-registration via FSL (https://fsl.fmrib.ox.ac.uk/fsl/) to allow for further comparisons. Following registration, the MP2RAGE images were processed through the fully automated pipeline of FreeSurfer 6.0 using the command “recon-all -all” (https://surfer.nmr.mgh.harvard.edu/). For each participant, the number of voxels and T_1_ values for the regions of interest (ROIs) were extracted using the Desikan–Killiany atlas in FreeSurfer. To assess the spatial similarity between the PI-MP2RAGE and CS-MP2RAGE segmentations for each ROI, the Dice Similarity Coefficient (DSC) was calculated based on the gray matter probability tissue masks.

### Statistical Analysis

PI-MP2RAGE and CS-MP2RAGE with undersampling factors of 3.8, 4.4, and 8.0 (samples per TR: 3.8 (250), 4.4 (252), and 8.0 (253)) were measured in ten participants. Additionally, an additional six participants were scanned under the same conditions as above, as well as under further CS-MP2RAGE conditions with undersampling factors of 3.8 (196 samples per TR), 8.0 (192 and 126 samples per TR), and 10.0 (126 samples per TR). Mean volumes and T_1_ values in each ROI were compared between PI-MP2RAGE and CS-MP2RAGE. Paired two-sided t-tests were used for comparison involving undersampling factors of 3.8 (250 samples per TR), 4.4 (252 samples per TR), and 8.0 (253 samples per TR) based on data from sixteen participants. Welch’s two-sided t-tests were used for comparisons involving undersampling factors of 3.8 (196 samples per TR), 8.0 (192 and 126 samples per TR), and 10.0 (126 samples per TR), comparing PI-MP2RAGE data from sixteen participants to CS-MP2RAGE data from six participants. Bonferroni correction was applied to adjust for multiple comparisons.

## Results

In this study, we systematically evaluated the effect of undersampling factors and sampling per TR on the quality of CS-MP2RAGE images compared with a reference dataset, PI-MP2RAGE, focusing on segmentation accuracy, voxel count consistency, and T_1_ quantitative accuracy. In CS-MP2RAGE, a larger undersampling factor and a greater number of samples per TR contributed to a shorter acquisition time.

**Fig 1:**
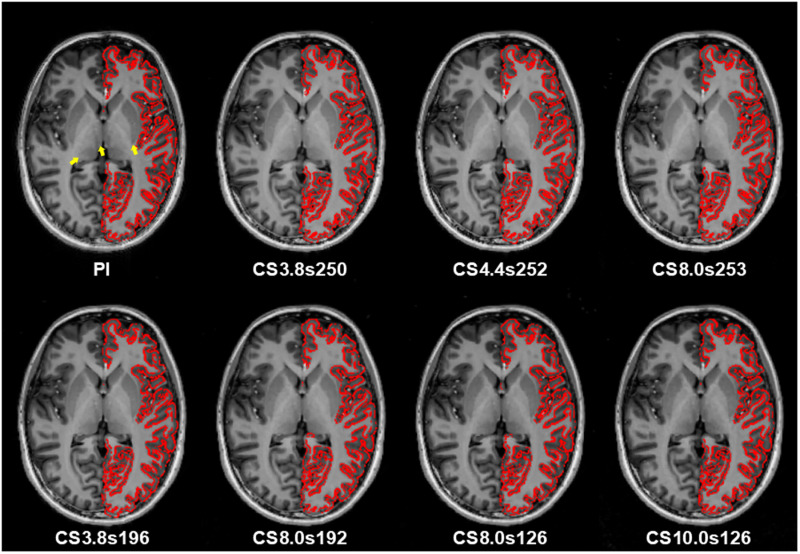
Comparison of T_1_-weighted images between PI-MP2RAGE and CS-MP2RAGE across all undersampling factors and samples per TR. Axial plane images from a representative subject are shown. The labels at the bottom of each image indicate the undersampling factors and the corresponding samples per TR. Red contour lines indicate gray matter segmentation in the left hemisphere.

Fig 1 shows the images obtained from PI-MP2RAGE and CS-MP2RAGE, along with undersampled and accelerated images. As the undersampling factor increases (upper row in Fig 1), the images appear progressively smoother and lose fine structural details, particularly in regions such as the basal ganglia and thalamus (arrows in Fig 1 and magnified view in S1 Fig 1). In addition, a clear thickening of the gray matter is observed in certain regions. In contrast, reducing the level of undersampling enhances the gray matter contrast and makes it similar to the PI-MP2RAGE image. In particular, this contrast improvement persists even at higher undersampling factors.

**Fig 2:**
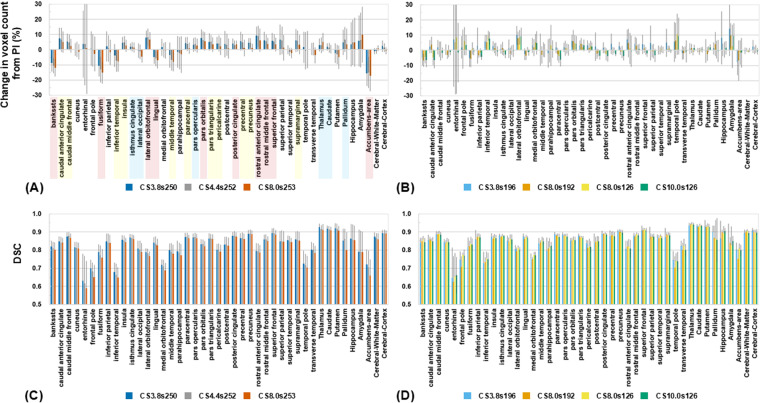
Comparison of voxel counts and segmentation overlap across brain regions between CS-MP2RAGE and PI-MP2RAGE. (A, B) Changes in voxel counts within each region of interest (ROI) based on the Desikan–Killiany atlas in FreeSurfer, comparing PI-MP2RAGE and CS-MP2RAGE. (A) Results for CS-MP2RAGE undersampling factors 3.8, 4.4, and 8.0, with corresponding samples per TR of 250, 252, and 253, respectively. (B) Results for CS-MP2RAGE undersampling factors 3.8, 8.0, 8.0, and 10.0, with samples per TR of 196, 192, 126, and 126, respectively. (C, D) DSCs for each ROI, representing the degree of segmentation overlap between PI-MP2RAGE and CS-MP2RAGE. (C) Results for CS-MP2RAGE undersampling factors 3.8, 4.4, and 8.0, with samples per TR of 250, 252, and 253, respectively. (D) Results for CS-MP2RAGE undersampling factors 3.8, 8.0, 8.0, and 10.0, with samples per TR of 196, 192, 126, and 126, respectively. In (A), the colors of the ROI names indicate the number of CS-MP2RAGE conditions that show statistically significant differences compared to PI-MP2RAGE: red for three, yellow for two, and blue for one.

To quantitatively assess these effects, ROIs were defined using the Desikan–Killiany atlas in FreeSurfer. Volumes, that is, voxel counts, within each ROI were compared across different reconstruction conditions to evaluate the effects of undersampling and acceleration on structural integrity and image quality. Fig 2 compares the regional volumes after segmenting each T_1_-weighted image using UNIDEN. As shown in Fig 2A, regional volumes were analyzed for images with relatively high samples per TR while varying the undersampling factor. In contrast, in Fig 2B, we examined regional volume variations as the samples per TR were reduced while varying the undersampling factor. Across all images, regional volume differences compared with PI-MP2RAGE remained within ±10% for most regions. However, higher undersampling factors resulted in greater volume variations than lower undersampling conditions. In most regions, higher accelerations tended to increase regional volumes. Conversely, when the number of samples per TR was reduced, the regional volume differences compared with PI-MP2RAGE were relatively small. Furthermore, as the number of samples per TR decreased, the extent of regional volume variation also diminished, indicating that fewer samples per TR contributed to maintaining structural consistency. The DSC was calculated to determine whether increased regional volume resulted in greater overlap between the PI-MP2RAGE and CS-MP2RAGE images (Figs 2C and 2D). In most regions, the DSC values exceeded 0.8, indicating a high degree of similarity between CS-MP2RAGE and PI-MP2RAGE images. However, images reconstructed with higher undersampling factors in CS-MP2RAGE, which showed greater volume increases than PI-MP2RAGE, tended to have lower DSC values than those reconstructed with lower undersampling factors, where volume changes were smaller. This suggests that fewer samples per TR resulted in smaller volume changes and higher spatial consistency between images. An important observation was that the entorhinal cortex showed considerable variability in volume changes compared with PI-MP2RAGE, resulting in relatively lower DSC values for all CS-MP2RAGE. One possible factor is that the image quality in deep regions, which are more susceptible to susceptibility effects owing to the characteristics of 7T MRI or its coils, may lead to suboptimal segmentation of PI images in some subjects. As a result, the volume variations increased, and the overlap between the images decreased. The DSC values in such regions should be interpreted cautiously, particularly when considering the influence of segmentation accuracy.

**Fig 3:**
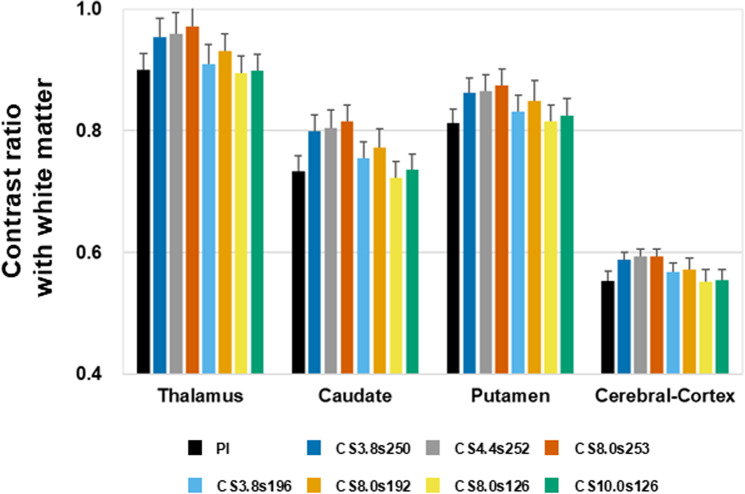
Contrast ratio between gray matter and white matter in PI-MP2RAGE and CS-MP2RAGE. Results of CS-MP2RAGE are shown for undersampling factors of 3.8, 4.4, 8.0, and 10.0, with corresponding samples per TR of 3.8 (250), 4.4 (252), 8.0 (253), 3.8 (196), 8.0 (192), 8.0 (126), and 10.0 (126).

To evaluate the contrast between gray matter (GM) and white matter (WM) in CS-MP2RAGE images, the GM/WM intensity ratio was analyzed (Fig 3). A lower ratio indicates higher contrast. Similar to the volume results, images with higher undersampling factors with similar samples per TR tended to show reduced contrast compared to PI-MP2RAGE. Conversely, decreasing the number of samples per TR with the same undersampling factor improved the contrast, making it more comparable to PI-MP2RAGE. Notably, even with an undersampling factor as high as 10, images reconstructed with fewer samples per TR maintained contrast levels equivalent to those of PI-MP2RAGE. These findings suggest that while high acceleration can affect image quality, maintaining a lower number of samples per TR is crucial for preserving the contrast between gray and white matter and ensuring consistency with PI-MP2RAGE.

**Fig 4:**
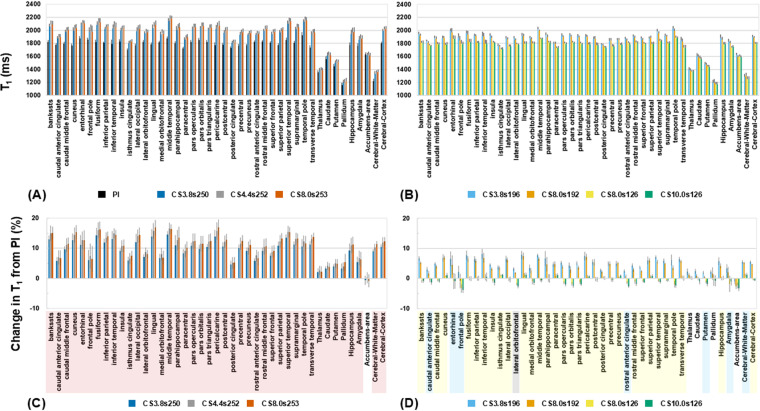
Comparison of T_1_ values across brain regions between CS-MP2RAGE and PI-MP2RAGE. (A, B) T_1_ values within each region of interest (ROI) based on the Desikan–Killiany atlas in FreeSurfer. (A) Results for PI-MP2RAGE and CS-MP2RAGE undersampling factors 3.8, 4.4, and 8.0, with corresponding samples per TR of 250, 252, and 253, respectively. (B) Results for CS-MP2RAGE undersampling factors 3.8, 8.0, 8.0, and 10.0, with samples per TR of 196, 192, 126, and 126, respectively. (C, D) Change in T_1_ within each ROI, comparing PI-MP2RAGE and CS-MP2RAGE. (C) Results for CS-MP2RAGE undersampling factors 3.8, 4.4, and 8.0, with samples per TR of 250, 252, and 253, respectively. (D) Results for CS-MP2RAGE undersampling factors 3.8, 8.0, 8.0, and 10.0, with samples per TR of 196, 192, 126, and 126, respectively. In (C) and (D), the colors of the ROI names indicate the number of CS-MP2RAGE conditions that show statistically significant differences compared to PI-MP2RAGE: gray for four, red for three, yellow for two, and blue for one.

**Fig 5:**
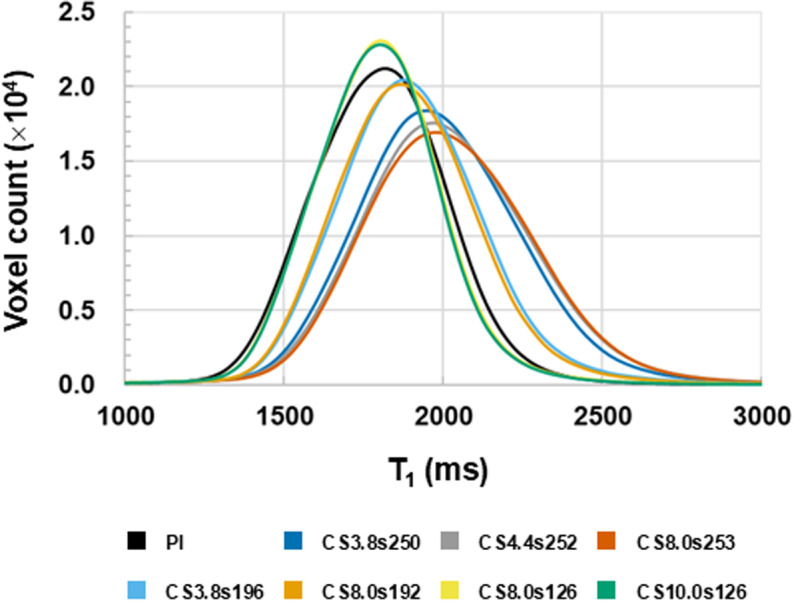
T_1_ distributions in PI-MP2RAGE and CS-MP2RAGE across the whole gray matter. T_1_ mean values were obtained in the whole gray matter over all subjects.

Finally, one of the key advantages of MP2RAGE is its ability to calculate T_1_ values based on images with two inversion times. To assess this, we analyzed the T_1_ values obtained from different MP2RAGE reconstructions. Figs 4A and 4B shows the T_1_ values for each region in all MP2RAGE images, whereas Figs 4C and 4D illustrate the differences in T_1_ values between PI-MP2RAGE and CS-MP2RAGE. When the undersampling factor was high, the T_1_ values showed significant deviations exceeding 10%, with a tendency to be overestimated compared with PI-MP2RAGE, which was statistically significant in all regions except for the accumbens. As the number of samples per TR decreased, the discrepancy also decreased. For example, when the undersampling factor was set to approximately 190, the T_1_ overestimation was reduced to approximately 5%; however, most regions were significant, except for the thalamic regions. When the number of samples per TR was lowered to 126, the T_1_ values closely matched those obtained using PI-MP2RAGE. Fig 5 illustrates these trends by showing voxel-wise T_1_ distributions for PI-MP2RAGE and different CS-MP2RAGE conditions. The corresponding standard deviation error bars are shown in S2 Fig. The peak positions and shapes of the T_1_ histograms revealed that higher undersampling factors tend to shift the T_1_ distribution toward longer values, while reducing the number of samples per TR mitigates this effect. Agreement with PI-MP2RAGE was observed when the number of samples per TR was small, suggesting that an optimized sampling strategy can minimize systematic T_1_ overestimation while maintaining the acceleration benefits of CS.

## Discussion

Our study is among the first to directly compare compressed sensing with parallel imaging in the MP2RAGE framework at UHF. The findings provide quantitative evidence of their relative and combined performances. This study evaluated the impact of undersampling factors and samples per TR on acquisition time, image quality, T_1_ mapping accuracy, and segmentation consistency in the CS-MP2RAGE sequence. Although increasing the undersampling factor significantly reduced acquisition time, it also resulted in quantitatively noticeable segmentation volume mismatch of up to 20% and increased T_1_ values, even though the image quality could not be qualitatively distinguished through visual inspection. However, reducing samples per TR increased acquisition time but improved image quality, allowing for higher undersampling factors without a significant loss in image quality. This setting did not result in the shortest scan time in this study but provided the optimal balance between acquisition speed and quantitative accuracy. Notably, two different combinations of low samples per TR and undersampling factors resulted in comparable performance. One of these combinations reduced scan time by up to 61% compared to the reference parallel imaging techniques such as GRAPPA, resulting in a total scan time of less than 3 minutes, while both combinations maintained minimal volume deviations, high Dice similarity coefficients, comparable gray–white matter contrast, and T_1_ values consistent with those of the reference. These findings highlight the potential of compressed sensing as an efficient method for accelerating MP2RAGE acquisition.

CS can significantly reduce acquisition time across various imaging contexts, with undersampling factors reaching up to 8 [8–11]. Trotier et al. (2022) [10] and Ferraro et al. (2022) [11] evaluated CS-MP2RAGE with MPRAGE for brain volume measurements at 3T. Trotier et al. (2022) [10] reported that CS-MP2RAGE, with an undersampling factor of 8 at 0.8 mm³ isotropic resolution, provided comparable morphological images and improved GM/WM contrast, with no significant differences in GM and WM volume measurements. However, deep gray matter structures (including the thalamus, putamen, and caudate) exhibited volumetric differences of up to 8.5% compared with MPRAGE while maintaining high repeatability. Meanwhile, Ferraro et al. (2022) [11] (undersampling factor = 4.6) reported DSCs > 0.70 for 93.5% of regions, confirming segmentation consistency. However, the accumbens region showed lower similarity coefficients (0.5–0.6), which is consistent with our findings and highlights the challenge of CS-based segmentation in small or complex regions. In a similar study comparing CS-MP2RAGE and PI-MP2RAGE, Mussard et al. (2020) [6] investigated CS-MP2RAGE at 3T using undersampling factors of 3–7 and samples per TR between 174 and 198, reporting a 57% reduction in scan time with an undersampling factor of 4 and 190 samples per TR while maintaining high image quality. The parameters used in this study (undersampling factor = 3.8, samples per TR = 196) were comparable to those in the previous study [6]. The results indicated no significant difference in volume change, consistent with earlier findings [6], and the DSCs were greater than 0.9 in our results. Similarly, Hubner et al. (2024) [8] focused on thalamic nuclei segmentation using CS-MP2RAGE at 3T, applying an undersampling factor of 4.6 with approximately 195 samples per TR, which reduced scan time from 8:52–3:40 [6] and resulted in a 5% increase in thalamic volume. This finding aligns with our results and suggests that higher undersampling factors may lead to slight volumetric expansion in the thalamus, regardless of field strength. These results indicate that while CS enables significant scan time reductions, its impact on volumetric accuracy varies across different brain structures, necessitating careful parameter optimization for robust clinical and research applications.

A key advantage of MP2RAGE is its ability to calculate T_1_ values using two inversion time images, thereby providing more robust quantitative measurements. Our study demonstrated that the T_1_ values obtained from all MP2RAGE images fell within the range of previously reported T_1_ values in CS-MP2RAGE, including 1,950 ms [9] and 2,132 ms [12] in the gray matter and 1,361–1,802 ms [13] in various gray matter regions. The observed differences in T_1_ values could be attributed to variations in MP2RAGE imaging parameters, segmentation inaccuracies caused by local image artifacts, and residual partial volume effects—particularly at tissue-CSF boundaries. Our imaging parameters were similar to those used by Metere et al. (2017) [13], suggesting that methodological differences have contributed to these variations. However, an important finding of this study is that increasing the undersampling factor resulted in higher T_1_ values, exceeding a 10% deviation from PI-MP2RAGE. In contrast, combining a higher undersampling factor with reduced samples per TR produced T_1_ estimates that closely matched those of PI-MP2RAGE, indicating a compensatory effect. In CS reconstruction, undersampling results in sparsely sampled k-space data, which requires sparsity constraints and iterative optimization for image reconstruction. As undersampling increases, the reconstruction process may overregularize the data, leading to signal attenuation and potentially biased T_1_ estimation. Strong noise suppression in CS reconstruction enhances the low spatial frequency components, resulting in a smoother signal decay curve, which may artificially correct signal attenuation and ultimately cause T_1_ values to be overestimated. However, reducing the number of samples per TR in CS improves T_1_ accuracy by increasing the number of fully sampled k-space data per repetition, minimizing artificial signal modification. This allows for a more accurate estimation of T_1_, consistent with the values obtained using PI-MP2RAGE. These findings highlight the importance of optimizing undersampling factors and sampling density to achieve an optimal balance between acceleration efficiency and accurate T_1_ quantification in CS-MP2RAGE.

This study has some limitations. Although it is useful for neuroscience research that this study was conducted only on healthy participants, it remains unclear whether the optimal parameters observed here can be directly applied to brains disorders such as tumors, cerebrovascular disease and neurodegenerative conditions, which may affect tissue contrast. For example, cerebrovascular disease and white matter hyperintensities may alter the contrast between gray and white matter, while advanced gray matter atrophy, including that associated with neurodegenerative diseases such as dementia, can lead to thinning of the cortical ribbon, widening of sulci, and increased partial volume effects. These factors may impair segmentation accuracy. Our results showed that both gray and white matter contrast and segmentation results depend on undersampling factors and samples per TR. Therefore, the optimal parameters obtained in this study, which balance scan time and image quality, may not be generalized to broader and more heterogeneous populations, especially in clinical applications involving pathological conditions. Further studies are needed to evaluate the applicability and robustness of the proposed parameters in such clinical situations.

Furthermore, CS reconstruction requires considerable computational resources and expertise, which may limit its application in clinical settings. Further research should focus on developing computationally efficient algorithms to facilitate widespread adoption in resource-constrained settings.

## Conclusion

This study demonstrated that CS could accelerate MP2RAGE imaging by reducing scan time by up to 60% (< 3 min) while maintaining image quality, segmentation accuracy, and T_1_-mapping fidelity comparable to PI-MP2RAGE. Increasing the undersampling factor significantly reduced acquisition time but introduced segmentation mismatches and T_1_ value deviations. In contrast, reducing the number of samples per TR improved image quality, enabling higher undersampling without significant degradation. This setting provided the optimal balance between acceleration and quantitative accuracy, although it was not the shortest scan time in this study. CS-MP2RAGE remained effective even with up to a 60% reduction in imaging time, making it a promising method for UHF MRI, where signal loss and contrast reduction present challenges. By addressing time constraints, CS enhances the feasibility of MP2RAGE in clinical and research applications. Future studies should optimize these parameters and explore their applicability across various imaging conditions and populations.

## Supporting information

S1 Fig Enlarged view of subcortical structures for comparison of T_1_-weighted images between PI-MP2RAGE and CS-MP2RAGE across all undersampling factors and samples per TR. Enlarged axial views of subcortical regions from the same subject are shown in Fig. 1. Images highlight the thalamus and basal ganglia areas to illustrate differences in structural detail across acquisition conditions. As the undersampling factor increases, fine anatomical features in these regions appear progressively smoothed and less distinct.
(TIF)

S2 Fig T_1_ value distributions with error bars for PI-MP2RAGE and each CS-MP2RAGE condition. T_1_ distributions in the whole gray matter for PI-MP2RAGE and each CS-MP2RAGE condition. Each plot shows the mean T_1_ values across subjects, with error bars representing the standard deviation. These plots use the same dataset as Fig. 5 and are presented individually to enable clearer visualization of variability, which would be difficult to interpret in a combined figure due to overlapping distributions.
(TIF)
