# Supplementary figures and images for: Shortening 7T MP2RAGE acquisition with compressed sensing: Evaluating quantitative accuracy and structural consistency
Source: PLoS One. 2025 Jun 16;20(6):e0325783. doi: 10.1371/journal.pone.0325783 (PMC12169513; doi:10.1371/journal.pone.0325783)

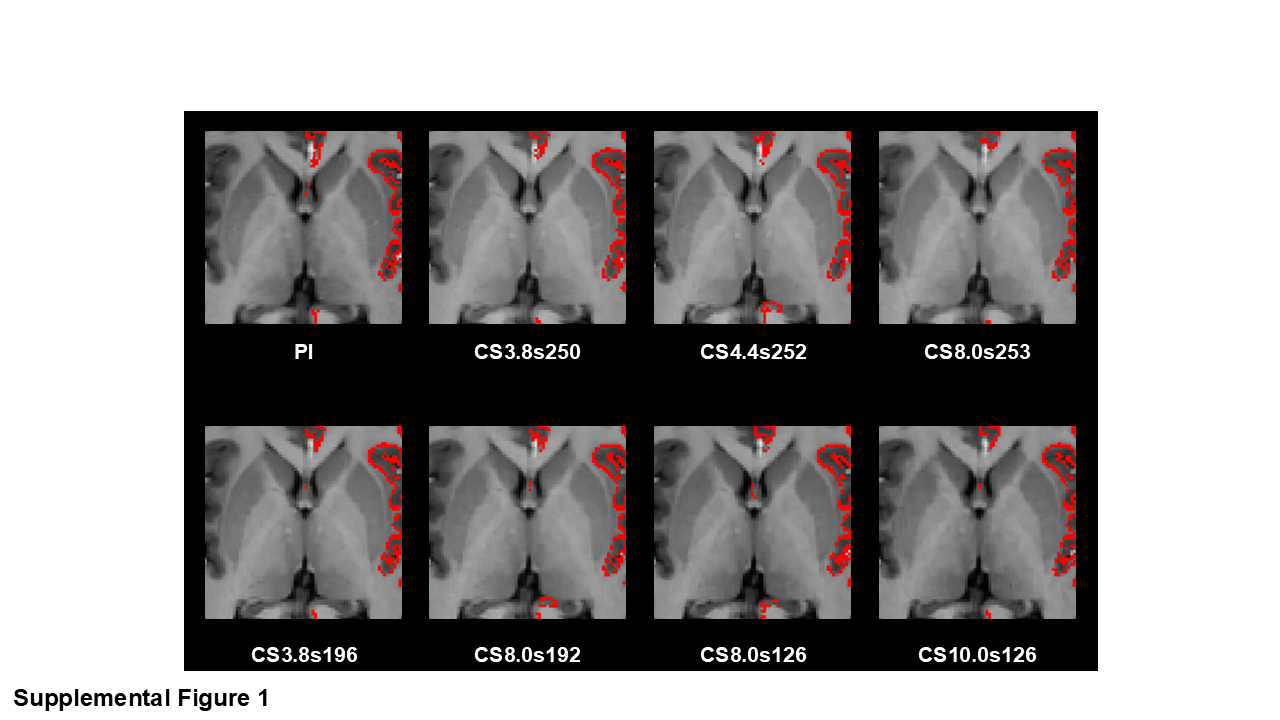

Supplement: S1 Fig — Enlarged axial views of subcortical regions from the same subject are shown in Fig. 1. Images highlight the thalamus and basal ganglia areas to illustrate differences in structural detail across acquisition conditions. As the undersampling factor increases, fine anatomical features in these regions appear progressively smoothed and less distinct. (TIF) [file pone.0325783.s001.tif]

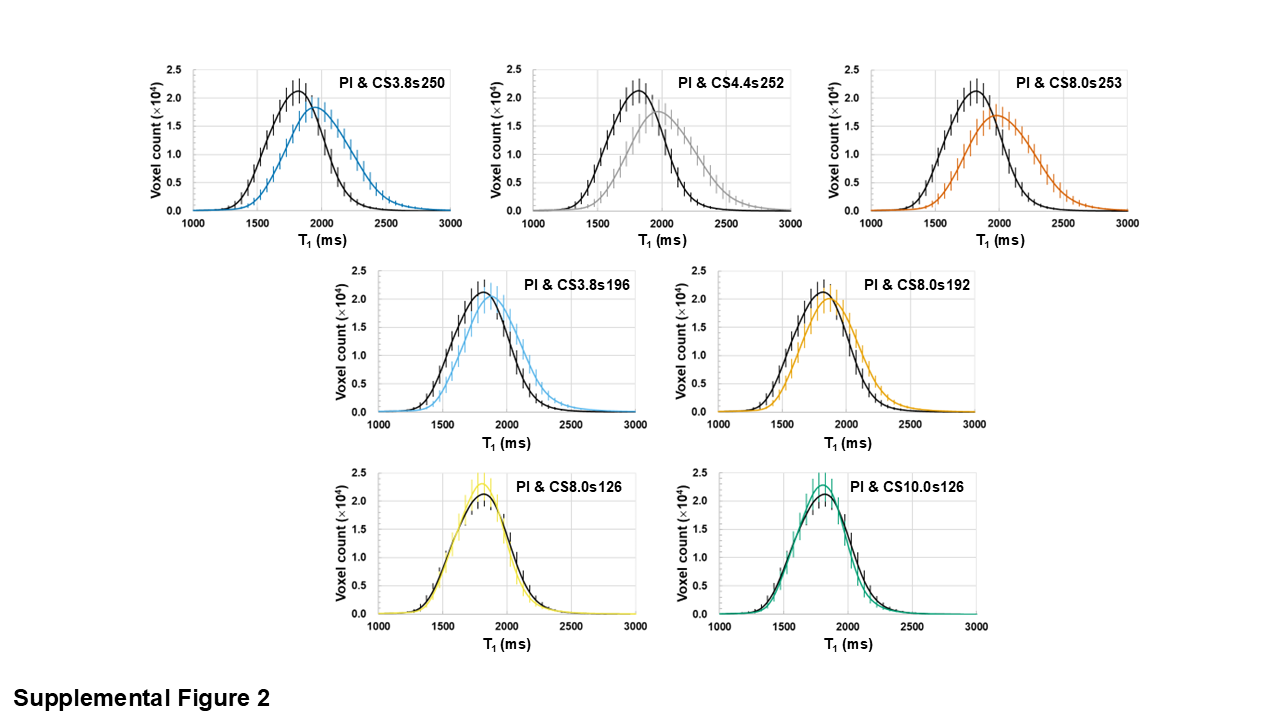

Supplement: S2 Fig — T1 distributions in the whole gray matter for PI-MP2RAGE and each CS-MP2RAGE condition. Each plot shows the mean T1 values across subjects, with error bars representing the standard deviation. These plots use the same dataset as Fig. 5 and are presented individually to enable clearer visualization of variability, which would be difficult to interpret in a combined figure due to overlapping distributions. (TIF) [file pone.0325783.s002.tif]
